# Hyperentanglement concentration of nonlocal two-photon six-qubit systems via the cross-Kerr nonlinearity

**DOI:** 10.1038/s41598-020-78529-2

**Published:** 2020-12-08

**Authors:** Qian Liu, Guo-Zhu Song, Tian-Hui Qiu, Xiao-Min Zhang, Hong-Yang Ma, Mei Zhang

**Affiliations:** 1 Research Center for Quantum Optics and Quantum Communication, School of Science, Qingdao University of Technology, Qingdao, 266525 China; 2 College of Physics and Materials Science, Tianjin Normal University, Tianjin, 300387 China; 3 Department of Physics, Applied Optics Beijing Area Major Laboratory, Beijing Normal University, Beijing, 100875 China

**Keywords:** Quantum information, Quantum mechanics

## Abstract

We present an efficient hyperentanglement concentration protocol (hyper-ECP) for two-photon six-qubit systems in nonlocal partially hyperentangled Bell states with unknown parameters. In our scheme, we use two identical partially hyperentangled states which are simultaneously entangled in polarization and two different longitudinal momentum degrees of freedom (DOFs) to distill the maximally hyperentangled Bell state. The quantum nondemolition detectors based on the cross-Kerr nonlinearity are used to realize the parity checks of two-photon systems in three DOFs. The hyper-ECP can extract all the useful entanglement source, and the success probability can reach the theory limit with the help of iteration. All these advantages make our hyper-ECP useful in long-distance quantum communication in the future.

## Introduction

As one of the striking features of quantum information, entanglement, has been widely used in quantum information processing, such as quantum teleportation^1^, controlled teleportation^2,3^, quantum dense coding^4,5^, quantum key distribution^6–9^, quantum secret sharing^10–12^, quantum state sharing^13,14^, quantum secure direct communication^15–20^, and so on. Single photons are interesting candidates for quantum communication, and they can carry quantum information in several degrees of freedom (DOFs). The entanglement in which photons are simultaneously entangled in more than one DOF could be called hyperentanglement. Many kinds of hyperentanglement have been discussed, such as polarization-momentum^21^, polarization-time-bin^22^, polarization-frequency^23^, and polarization-orbital-angular-momentum^24^. The hyperentangled Bell states in both the polarization and two different longitudinal momentum modes DOFs have been introduced in experiments^25,26^. Hyperentanglement of photon system can increase both the channel capacity of long-distance quantum communication^24^and its security. It can also be used on complete Bell states analysis^21,22,27,28^, high-speed quantum computation^29,30^, superdense coding^31^, quantum key distribution^32^, etc.

However, the entanglement and the fidelity of the entangled systems inevitably degrade because of the interaction with the environment during the storage and transmission. One of the methods to depress the noise effect on entangled systems is entanglement concentration. It can be used to extract the maximally entangled state from a large number of less entangled pure states. In 1996, Bennett *et al.*^33^ proposed the first entanglement concentration protocol (ECP) for two-photon system, which is called as Schmidt projection method. They utilized collective measurement to obtain the coefficient information. As it requires the collective measurement on multiple particles simultaneously, it is difficult to manipulate in experiment. Later, Bose *et al.*^34^ designed an efficient ECP with entanglement swapping. In 2000, Shi *et al.*^35^ proposed an ECP based on entanglement swapping and collective two-qubit unitary evolution. In 2001, Yamamoto *et al.*^36^ and Zhao *et al.*^37^ independently presented two ECPs assisted by linear-optical elements and postselection. In 2008, Sheng *et al.*^38^ presented an interesting ECP that exploited cross-Kerr nonlinearity, which had a higher efficiency and yield than those with linear optical elements with the help of iteration. Motivated by those innovation works, many interesting ECPs have been presented and discussed for different physical systems and different entangled states^39–48^.

The concentration of hyperentangled states also has been extensively studied in recent years. In 2013, Ren, Du, and Deng^49^ gave the first hyperentanglement concentration protocol (hyper-ECP) for two-photon four-qubit systems with linear optics. In 2014, Ren and Long^50^ proposed another hyper-ECP for nonlocal partially hyperentangled Bell states in polarization-spatial mode DOFs assisted by nonlinear interactions. In 2015, Li and Ghose^51^ presented two hyper-ECPs for time-bin and polarization hyperentangled states with unknown parameters and known parameters, respectively. In 2016, Cao *et al.*^52^ presented a hyper-ECP utilizing photonic module system. In 2017, Wang and Ren *et al.* gave two efficient hyper-ECPs for polarization-spatial-time-bin hyperentangled two-photon six-qubit systems^53,54^. The hyper-ECP for three-photon partially hyperentangled GHZ states in polarization, spatial-mode and time-bin DOFs with linear optics was also proposed in the next year^55^. The hyper-ECP for polarization-spatial-time-bin hyperentangled Bell states using cross-Kerr nonlinearity has also been discussed^56^. Although many hyper-ECPs have been presented, the concentration of two-photon six-qubit hyperentangled Bell states in both the polarization and the double longitudinal momentum modes DOFs has not been researched.

In this article, we present an efficient hyper-ECP for partially hyperentangled Bell states of two-photon six-qubit systems. Two photons considered in our hyper-ECP are simultaneously entangled in polarization and two longitudinal momentum DOFs, which is not taken into account in other hyper-ECPs. We use the quantum nondemolition detectors (QNDs) to check the parity of the two-photon in three DOFs to implement our protocol, and therefore the unsuccessful instances in each round can be reused in the next concentration round. The success probability in our scheme can be greatly improved and the maximum of the success probability is nearly 100% with the help of iteration. Moreover, it does not require that the parties know the exact information about the partially hyperentangled Bell states. These good features make our scheme efficient and useful for quantum information processing involving hyperentanglement.

## Results

*The hyper-ECP process for two-photon six-qubit systems* The hyperentangled Bell state of two-photon six-qubit systems in three DOFs can be described as follows^26^:

1 $$\begin{aligned} |HE_{6}\rangle= & {} \frac{1}{\sqrt{2}}(|H\rangle _{A}|H\rangle _{B} +|V\rangle _{A}|V\rangle _{B})\otimes \frac{1}{\sqrt{2}} (|l\rangle _{A}|r\rangle _{B}+|r\rangle _{A}|l\rangle _{B})\nonumber \\&\otimes \frac{1}{\sqrt{2}} (|I\rangle _{A}|I\rangle _{B}+|E\rangle _{A}|E\rangle _{B}). \end{aligned}$$

Here the subscripts A and B denote the two photons. The three independent DOFs are polarization (*H*/*V*) and a double longitudinal momentum (*r*/*l* and *E*/*I*). *H* and *V* represent the horizontal and vertical polarization of photons, respectively. *l* (*r*) represents the left (right) mode and *E* (*I*) represents the external (internal) mode. The four Bell states in the polarization DOF of two-photon systems can be written as

2 $$\begin{aligned} |\phi ^{\pm }\rangle ^{P}_{AB}= & {} \frac{1}{\sqrt{2}}(|H\rangle _{A} |H\rangle _{B}\pm |V\rangle _{A}|V\rangle _{B}), \end{aligned}$$

3 $$\begin{aligned} |\psi ^{\pm }\rangle ^{P}_{AB}= & {} \frac{1}{\sqrt{2}}(|H\rangle _{A} |V\rangle _{B}\pm |V\rangle _{A}|H\rangle _{B}), \end{aligned}$$

and four Bell states in the first longitudinal momentum DOF are

4 $$\begin{aligned} |\phi ^{\pm }\rangle ^{F}_{AB}= & {} \frac{1}{\sqrt{2}}(|l\rangle _{A} |l\rangle _{B}\pm |r\rangle _{A}|r\rangle _{B}), \end{aligned}$$

5 $$\begin{aligned} |\psi ^{\pm }\rangle ^{F}_{AB}= & {} \frac{1}{\sqrt{2}}(|l\rangle _{A} |r\rangle _{B}\pm |r\rangle _{A}|l\rangle _{B}), \end{aligned}$$

while the four Bell states in the second longitudinal momentum DOF can be denoted as

6 $$\begin{aligned} |\phi ^{\pm }\rangle ^{S}_{AB}= & {} \frac{1}{\sqrt{2}}(|I\rangle _{A} |I\rangle _{B}\pm |E\rangle _{A}|E\rangle _{B}), \end{aligned}$$

7 $$\begin{aligned} |\psi ^{\pm }\rangle ^{S}_{AB}= & {} \frac{1}{\sqrt{2}}(|I\rangle _{A} |E\rangle _{B}\pm |E\rangle _{A}|I\rangle _{B}). \end{aligned}$$

Here $|\phi ^{\pm }\rangle ^{i}_{AB}$ ($i=P,F,S$) is the even-parity state of photons in *i* DOF, while $|\psi ^{\pm }\rangle ^{j}_{AB}$ ($j=P,F,S$) is the odd-parity state of photons in *j* DOF, the superscripts *P*, *F*, and *S* denote the polarization, the first longitudinal momentum, and the second longitudinal momentum DOFs of a two-photon six-qubit system, respectively. In long-distance quantum communication, the maximally hyperentangled Bell state $|HE_{6}\rangle$ may decay to a partially hyperentangled Bell state $|\psi \rangle _{AB}$ after passing through the noisy channels. Here,

8 $$\begin{aligned} |\psi \rangle _{AB}= & {} (\alpha |H\rangle _{A}|H\rangle _{B}+\beta |V\rangle _{A}|V\rangle _{B}) \otimes (\gamma |l\rangle _{A}|r\rangle _{B}+\delta |r\rangle _{A}|l\rangle _{B})\nonumber \\&\otimes (\epsilon |I\rangle _{A}|I\rangle _{B}+\varepsilon |E\rangle _{A}|E\rangle _{B}). \end{aligned}$$

The parameters $\alpha$, $\beta$, $\gamma$, $\delta$, $\epsilon$, $\varepsilon$ are unkown and satisfy the normalization condition $|\alpha |^{2}+|\beta |^{2}=|\gamma |^{2}+|\delta |^{2}=|\epsilon |^{2}+|\varepsilon |^{2}=1$.

**Figure 1:**
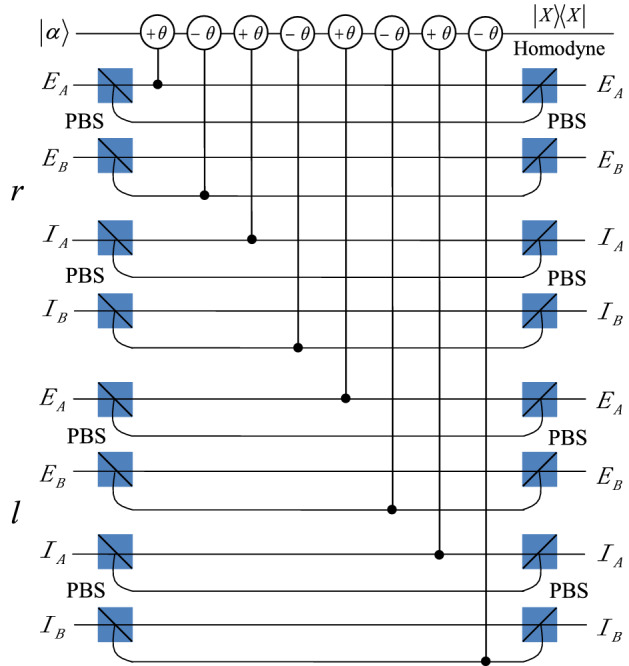
Schematic diagram of the principle of P-QND constructed by the cross-Kerr nonlinearity, one can distinguish the states $|H\rangle _{A}|H\rangle _{B}$ and $|V\rangle _{A}|V\rangle _{B}$ from the states $|H\rangle _{A}|V\rangle _{B}$ and $|V\rangle _{A}|H\rangle _{B}$ with different phase shifts $\pm \theta$ and 0 of the coherent state, respectively. $|X\rangle \langle X|$ is the homodyne measurement to distinguish different phase shifts of the coherent probe beam. PBS denotes the polarizing beam splitter which is used to reflect the vertical (*V*) polarization photon and transmit the horizontal (*H*) polarization photon, respectively.

**Figure 2:**
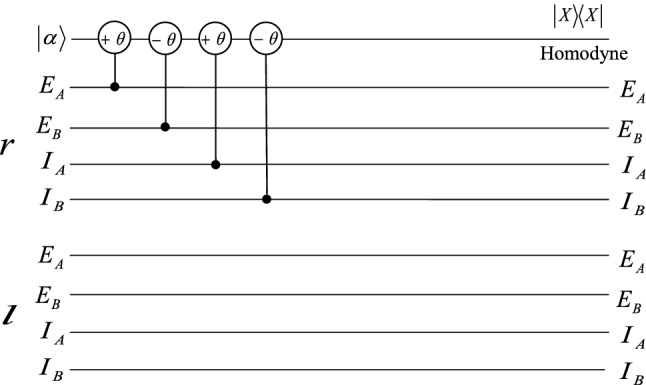
Schematic diagram of the principle of the F-QND. One can distinguish the states $|rr\rangle$ and $|ll\rangle$ from $|lr\rangle$ and $|rl\rangle$ by the different phase shifts 0 and $\pm \theta$ of the coherent state.

**Figure 3:**
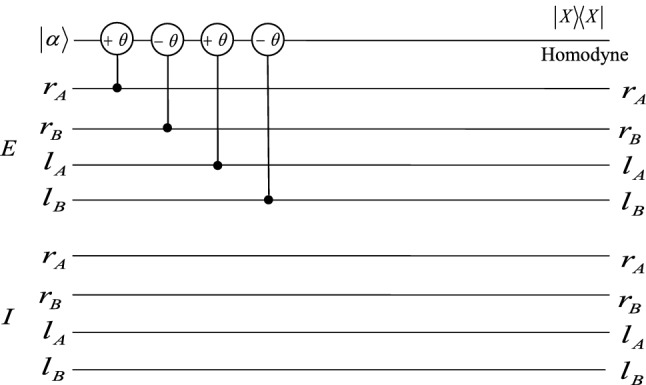
Schematic diagram of S-QND, which is used to distinguish the states $|II\rangle$ and $|EE\rangle$ from $|IE\rangle$ and $|EI\rangle$ by different phase shifts 0 and $\pm \theta$ of the coherent state.

In order to realize the hyperentanglement concentration of the unknown partially hyperentangled Bell state $|\psi \rangle _{AB}$, we use the QNDs shown in Figs. 1, 2, and 3. The P-QND shown in Fig. 1 has been described in Ref.^57^, and the other two QNDs are improved based on the schemes shown in Ref.^57^. Based on the principle of the cross-Kerr effect (see the Methods section), if one let the two photons A and B pass through the circuit as P-QND shown in Fig. 1, they can only get two measurement outcomes $|\alpha \rangle$ and $|\alpha e^{\pm i\theta }\rangle$ for the coherent probe beam, with the corresponding polarization states $|H\rangle _{A}|H\rangle _{B}$ ($|V\rangle _{A}|V\rangle _{B}$) and $|H\rangle _{A}|V\rangle _{B}$ ($|V\rangle _{A}|H\rangle _{B}$). It is essentially the polarization parity-check measurement of photons, which can be used to distinguish the even-parity states $|\phi ^{\pm }\rangle _{P}$ from the odd-parity states $|\psi ^{\pm }\rangle _{P}$. F-QND is the first longitudinal momentum parity-check QND, which can be used to distinguish the first longitudinal momentum states $|r\rangle _{A}|r\rangle _{B}$ ($|l\rangle _{A}|l\rangle _{B}$) from $|r\rangle _{A}|l\rangle _{B}$ ($|l\rangle _{A}|r\rangle _{B}$) by different phase shifts 0 and $\pm \theta$ of the coherent state. The phase shift of the coherent state for the second longitudinal momentum states $|I\rangle _{A}|I\rangle _{B}$ and $|E\rangle _{A}|E\rangle _{B}$ is 0, different from phase shift $\pm \theta$ of the states $|I\rangle _{A}|E\rangle _{B}$ and $|E\rangle _{A}|I\rangle _{B}$, which can be realized in S-QND, the second longitudinal momentum parity-check QND.

**Figure 4:**
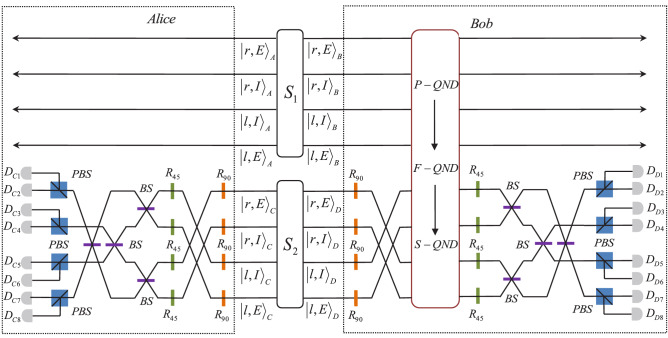
Schematic diagram of our hyper-ECP for a two-photon six-qubit partially hyperentangled Bell state with unknown parameters resorting to cross-Kerr nonlinearity. $S_{1}$ and $S_{2}$ are two identical partial hyperentanglement sources. $R_{90}$ and $R_{45}$ are half-wave plates, which are used to rotate the polarization of the state by $90^{o}$ and $45^{o}$, respectively. The 50:50 BS is used to accomplish the Hadamard operation for the two longitudinal momentum modes. PBS denotes a polarizing beam splitter which is used to transmit the horizontal polarization component and reflect the vertical polarization component. *D* represents the single-photon detector.

The basic principle of our hyper-ECP for two-photon six-qubit systems in an unknown partially hyperentangled state is shown in Fig. 4. Two photon pairs AB and CD shared by Alice and Bob can be in an identical partially hyperentangled state when they were produced from the same source and passed through the same channel. Thus the state of the photons CD can be denoted as

9 $$\begin{aligned} |\psi \rangle _{CD}= & {} (\alpha |H\rangle _{C}|H\rangle _{D}+\beta |V\rangle _{C}|V\rangle _{D}) \otimes (\gamma |l\rangle _{C}|r\rangle _{D}+\delta |r\rangle _{C}|l\rangle _{D})\nonumber \\&\otimes (\epsilon |I\rangle _{C}|I\rangle _{D}+\varepsilon |E\rangle _{C}|E\rangle _{D}). \end{aligned}$$

Before the photons CD are sent to Alice and Bob, the bit-flip operations are performed on the polarization mode of the photons by the half-wave plates $R_{90}$. The bit-flip operation of the double longitudinal momentum modes of photon C can be achieved by the exchange of the modes $|r,E\rangle _{C}$ and $|l,I\rangle _{C}$, $|r,I\rangle _{C}$ and $|l,E\rangle _{C}$, respectively. The bit-flip operation of the double longitudinal momentum modes of photon D can be achieved by the same way. Then Bob lets the photons B and D pass through the P-QND, F-QND and S-QND, successively. After the operations above, the whole state of the four photons and three coherent states can evolve into

10 $$\begin{aligned}&|\Psi \rangle _{ABCD}|\alpha \rangle _{1}|\alpha \rangle _{2}|\alpha \rangle _{3}\nonumber \\&\quad \rightarrow [(\alpha ^{2}|H\rangle _{A}|V\rangle _{C}|H\rangle _{B}|V\rangle _{D} +\beta ^{2}|V\rangle _{A}|H\rangle _{C}|V\rangle _{B}|H\rangle _{D})|\alpha \rangle _{1}\nonumber \\&\quad +\alpha \beta (|H\rangle _{A}|H\rangle _{C}|H\rangle _{B}|H\rangle _{D}|\alpha e^{i\theta }\rangle _{1}+|V\rangle _{A}|V\rangle _{C}|V\rangle _{B}|V\rangle _{D}|\alpha e^{-i\theta }\rangle _{1})]\nonumber \\&\quad \otimes [\gamma ^{2}|l\rangle _{A}|r\rangle _{C}|r\rangle _{B}|l\rangle _{D}|\alpha e^{i\theta }\rangle _{2} +\delta ^{2}|r\rangle _{A}|l\rangle _{C}|l\rangle _{B}|r\rangle _{D}|\alpha e^{-i\theta }\rangle _{2}\nonumber \\&\quad +\gamma \delta (|l\rangle _{A}|l\rangle _{C}|r\rangle _{B}|r\rangle _{D}|\alpha \rangle _{2} +|r\rangle _{A}|r\rangle _{C}|l\rangle _{B}|l\rangle _{D})|\alpha \rangle _{2}]\nonumber \\&\quad \otimes [\epsilon ^{2}|I\rangle _{A}|E\rangle _{C}|I\rangle _{B}|E\rangle _{D}|\alpha e^{-i\theta }\rangle _{3} +\varepsilon ^{2}|E\rangle _{A}|I\rangle _{C}|E\rangle _{B}|I\rangle _{D}|\alpha e^{i\theta }\rangle _{3}\nonumber \\&\quad +\epsilon \varepsilon (|I\rangle _{A}|I\rangle _{C}|I\rangle _{B}|I\rangle _{D} +|E\rangle _{A}|E\rangle _{C}|E\rangle _{B}|E\rangle _{D})|\alpha \rangle _{3}]. \end{aligned}$$

The corresponding relation between the states of ABCD, the parity check measurement results of three QNDs and the probability $P_{i}^{(1)}$ ($i=1, 2, 3, 4, 5, 6, 7, 8$) is shown in Table 1. The superscript “(1)” denotes the first round of concentration. According to the Homodyne measurement results of three QNDs, the eight collapsed states of four-photon system can be divided into four cases.

**Table 1:** The relation between the states of four photons, the parity check measurement results and the probability.

State of ABCD	P-QND	F-QND	S-QND	$P_{i}^{(1)}$
$|\Psi _{1}\rangle _{ABCD}$	Even	Even	Even	$8|\alpha \beta \gamma \delta \epsilon \varepsilon |^{2}$
$|\Psi _{2}\rangle _{ABCD}$	Odd	Even	Even	$4|\gamma \delta \epsilon \varepsilon |^{2}(|\alpha |^{4}+|\beta |^{4})$
$|\Psi _{3}\rangle _{ABCD}$	Even	Odd	Even	$4|\alpha \beta \epsilon \varepsilon |^{2}(|\gamma |^{4}+|\delta |^{4})$
$|\Psi _{4}\rangle _{ABCD}$	Even	Even	Odd	$4|\alpha \beta \gamma \delta |^{2}(|\epsilon |^{4}+|\varepsilon |^{4})$
$|\Psi _{5}\rangle _{ABCD}$	Odd	Odd	Even	$2|\epsilon \varepsilon |^{2}(|\alpha |^{4}+|\beta |^{4})(|\gamma |^{4}+|\delta |^{4})$
$|\Psi _{6}\rangle _{ABCD}$	odd	even	odd	$2|\gamma \delta |^{2}(|\alpha |^{4}+|\beta |^{4})(|\epsilon |^{4}+|\varepsilon |^{4})$
$|\Psi _{7}\rangle _{ABCD}$	even	odd	odd	$2|\alpha \beta |^{2}(|\gamma |^{4}+|\delta |^{4})(|\epsilon |^{4}+|\varepsilon |^{4})$
$|\Psi _{8}\rangle _{ABCD}$	odd	odd	odd	$(|\alpha |^{4}+|\beta |^{4})(|\gamma |^{4}+|\delta |^{4})(|\epsilon |^{4}+|\varepsilon |^{4})$

In the first case, all the three parity check measurements give even-parity results. The remaining state of the four-photon system can be described as

11 $$\begin{aligned} |\Psi _{1}\rangle _{ABCD}= & {} \frac{\alpha \beta }{\sqrt{P_{1}^{(1)}}}(|H\rangle _{A} |H\rangle _{C}|H\rangle _{B}|H\rangle _{D} +|V\rangle _{A}|V\rangle _{C}|V\rangle _{B}|V\rangle _{D})\nonumber \\&\otimes \gamma \delta (|l\rangle _{A}|l\rangle _{C}|r\rangle _{B}|r\rangle _{D} +|r\rangle _{A}|r\rangle _{C}|l\rangle _{B}|l\rangle _{D})\nonumber \\&\otimes \epsilon \varepsilon (|I\rangle _{A}|I\rangle _{C}|I\rangle _{B}|I\rangle _{D} +|E\rangle _{A}|E\rangle _{C}|E\rangle _{B}|E\rangle _{D}). \end{aligned}$$

The probability that Alice and Bob get the above state is $P_{1}^{(1)}=8|\alpha \beta \gamma \delta \epsilon \varepsilon |^{2}$.

Alice and Bob use $R_{45}$ to rotate the polarization of the state by $45^{\circ }$. BSs are used to perform the Hadamard operation on the double longitudinal momentum DOFs of the state. Then the selected term shown in Eq. (11) is transformed into

12 $$\begin{aligned} |\Psi ^{\prime }_{1}\rangle _{ABCD}= & {} \frac{1}{\sqrt{2}}(|\phi ^{+} \rangle ^{P}_{AB}|\phi ^{+}\rangle ^{P}_{CD}+|\phi ^{-}\rangle ^{P}_{AB}| \psi ^{+}\rangle ^{P}_{CD})\nonumber \\&\otimes \frac{1}{\sqrt{2}}(|\psi ^{+}\rangle ^{F}_{AB}|\phi ^{-} \rangle ^{F}_{CD}+|\psi ^{-}\rangle ^{F}_{AB}|\psi ^{-}\rangle ^{F}_{CD})\nonumber \\&\otimes \frac{1}{\sqrt{2}}(|\phi ^{+}\rangle ^{S}_{AB}|\phi ^{+} \rangle ^{S}_{CD}+|\phi ^{-}\rangle ^{S}_{AB}|\psi ^{+}\rangle ^{S}_{CD}). \end{aligned}$$

**Table 2:** The relation between the states of photon pair CD and the measurement results.

States of CD	Detectors
$|\phi ^{\pm }\rangle ^{P}_{CD}|\phi ^{\pm }\rangle ^{F}_{CD}|\phi ^{\pm }\rangle ^{S}_{CD}$	$D_{C1}D_{D1}$ $D_{C2}D_{D2}$ $D_{C3}D_{D3}$ $D_{C4}D_{D4}$
	$D_{C5}D_{D5}$ $D_{C6}D_{D6}$ $D_{C7}D_{D7}$ $D_{C8}D_{D8}$
$|\phi ^{\pm }\rangle ^{P}_{CD}|\phi ^{\pm }\rangle ^{F}_{CD}|\psi ^{\pm }\rangle ^{S}_{CD}$	$D_{C1}D_{D3}$ $D_{C3}D_{D1}$ $D_{C6}D_{D8}$ $D_{C8}D_{D6}$
	$D_{C2}D_{D4}$ $D_{C4}D_{D2}$ $D_{C5}D_{D7}$ $D_{C7}D_{D5}$
$|\phi ^{\pm }\rangle ^{P}_{CD}|\psi ^{\pm }\rangle ^{F}_{CD}|\phi ^{\pm }\rangle ^{S}_{CD}$	$D_{C2}D_{D7}$ $D_{C4}D_{D5}$ $D_{C5}D_{D4}$ $D_{C7}D_{D2}$
	$D_{C1}D_{D8}$ $D_{C3}D_{D6}$ $D_{C6}D_{D3}$ $D_{C8}D_{D1}$
$|\phi ^{\pm }\rangle ^{P}_{CD}|\psi ^{\pm }\rangle ^{F}_{CD}|\psi ^{\pm }\rangle ^{S}_{CD}$	$D_{C2}D_{D5}$ $D_{C4}D_{D7}$ $D_{C5}D_{D2}$ $D_{C7}D_{D4}$
	$D_{C1}D_{D6}$ $D_{C3}D_{D8}$ $D_{C6}D_{D1}$ $D_{C8}D_{D3}$
$|\psi ^{\pm }\rangle ^{P}_{CD}|\psi ^{\pm }\rangle ^{F}_{CD}|\phi ^{\pm }\rangle ^{S}_{CD}$	$D_{C1}D_{D2}$ $D_{C3}D_{D4}$ $D_{C6}D_{D5}$ $D_{C8}D_{D7}$
	$D_{C2}D_{D1}$ $D_{C4}D_{D3}$ $D_{C5}D_{D6}$ $D_{C7}D_{D8}$
$|\psi ^{\pm }\rangle ^{P}_{CD}|\psi ^{\pm }\rangle ^{F}_{CD}|\psi ^{\pm }\rangle ^{S}_{CD}$	$D_{C2}D_{D3}$ $D_{C4}D_{D1}$ $D_{C5}D_{D8}$ $D_{C7}D_{D6}$
	$D_{C1}D_{D4}$ $D_{C3}D_{D2}$ $D_{C6}D_{D7}$ $D_{C8}D_{D5}$
$|\psi ^{\pm }\rangle ^{P}_{CD}|\psi ^{\pm }\rangle ^{F}_{CD}|\phi ^{\pm }\rangle ^{S}_{CD}$	$D_{C1}D_{D7}$ $D_{C4}D_{D6}$ $D_{C5}D_{D3}$ $D_{C7}D_{D1}$
	$D_{C2}D_{D8}$ $D_{C3}D_{D5}$ $D_{C6}D_{D4}$ $D_{C8}D_{D2}$
$|\psi ^{\pm }\rangle ^{P}_{CD}|\psi ^{\pm }\rangle ^{F}_{CD}|\psi ^{\pm }\rangle ^{S}_{CD}$	$D_{C1}D_{D5}$ $D_{C3}D_{D7}$ $D_{C6}D_{D2}$ $D_{C8}D_{D4}$
	$D_{C2}D_{D6}$ $D_{C4}D_{D8}$ $D_{C5}D_{D1}$ $D_{C7}D_{D3}$

The last step is to distinguish the photons C and D in different polarization and different longitudinal momentum DOFs. The PBSs are used to transmit the horizontal polarization component and reflect the vertical polarization component. The corresponding relation between the measurement results and the states of CD is shown in Table 2. From Table 2, one can see that if both the single-photon detectors $D_{C1}$ and $D_{D1}$ click, the photon pair AB is left in the state

13 $$\begin{aligned} |\psi _{f}\rangle _{AB}=|\phi ^{+}\rangle ^{P}_{AB}|\psi ^{+} \rangle ^{F}_{AB}|\phi ^{+}\rangle ^{S}_{AB}. \end{aligned}$$

That is, the two-photon system AB is projected into the ideal maximally hyperentangled Bell state. Finally, according to the results of the measurement, one can perform corresponding phase-flip operations on the qubits to achieve the ideal state. The phase-flip operations on the qubits can be accomplished by putting conditional half-wave plates in the appropriate paths of photon. The corresponding relation between the final collapsed hyperentangled Bell states of photon pair AB, the half-wave plates and the relevant paths of photon is shown in Table 3. $R_{0}$ denotes the half-wave plate set at $0^{o}$ which performs the operation $|H\rangle \rightarrow |H\rangle$, $|V\rangle \rightarrow -|V\rangle$ on the polarization mode of photons. The half-wave plates $R_{90}$ performs the bit-flip operation on the polarization mode of the photons. If the photon pair AB is in the hyperentangled Bell state $|\phi ^{-}\rangle ^{P}_{AB}|\psi ^{-}\rangle ^{F}_{AB}|\phi ^{-}\rangle ^{S}_{AB}$, Alice can accomplish the phase-flip operations for all the three modes by putting $R_{0}$ in the paths $|l,I\rangle$ and $|r,E\rangle$, $R_{90}$, $R_{0}$ in the paths $|r,I\rangle$ and $|l,E\rangle$ of the photon A, respectively.

**Table 3:** The relation between the states of photon pair AB , the half-wave plates and the relevant paths.

State of AB	Half-wave plates	Paths
$|\phi ^{+}\rangle ^{P}_{AB}|\psi ^{+}\rangle ^{F}_{AB}|\phi ^{+}\rangle ^{S}_{AB}$	none	none
$|\phi ^{+}\rangle ^{P}_{AB}|\psi ^{+}\rangle ^{F}_{AB}|\phi ^{-}\rangle ^{S}_{AB}$	$R_{0}, R_{90}, R_{0}$	$|r,E\rangle$, $|l,E\rangle$
$|\phi ^{+}\rangle ^{P}_{AB}|\psi ^{-}\rangle ^{F}_{AB}|\phi ^{+}\rangle ^{S}_{AB}$	$R_{0}, R_{90}, R_{0}$	$|r,I\rangle$, $|r,E\rangle$
$|\phi ^{+}\rangle ^{P}_{AB}|\psi ^{-}\rangle ^{F}_{AB}|\phi ^{-}\rangle ^{S}_{AB}$	$R_{0}, R_{90}, R_{0}$	$|r,I\rangle$, $|l,E\rangle$
$|\phi ^{-}\rangle ^{P}_{AB}|\psi ^{+}\rangle ^{F}_{AB}|\phi ^{+}\rangle ^{S}_{AB}$	$R_{0}$	all the four paths
$|\phi ^{-}\rangle ^{P}_{AB}|\psi ^{+}\rangle ^{F}_{AB}|\phi ^{-}\rangle ^{S}_{AB}$	$R_{0}$	$|r,I\rangle$, $|l,I\rangle$
	$R_{90}, R_{0}$	$|r,E\rangle$, $|l,E\rangle$
$|\phi ^{-}\rangle ^{P}_{AB}|\psi ^{-}\rangle ^{F}_{CD}|\phi ^{+}\rangle ^{S}_{AB}$	$R_{0}$	$|l,I\rangle$, $|l,E\rangle$
	$R_{90}, R_{0}$	$|r,I\rangle$, $|r,E\rangle$
$|\phi ^{-}\rangle ^{P}_{AB}|\psi ^{-}\rangle ^{F}_{AB}|\phi ^{-}\rangle ^{S}_{AB}$	$R_{0}$	$|l,I\rangle$, $|r,E\rangle$
	$R_{90}, R_{0}$	$|r,I\rangle$, $|l,E\rangle$

In the second case, one of the parity check measurement results is odd-parity result. The four-photon system is projected into the state $|\Psi _{2}\rangle _{ABCD}$, $|\Psi _{3}\rangle _{ABCD}$ or $|\Psi _{4}\rangle _{ABCD}$, respectively, with the corresponding probability $P_{i}^{(1)}$ ($i=2,3,4$). We take the state $|\Psi _{2}\rangle _{ABCD}$ as an example. Here

14 $$\begin{aligned} |\Psi _{2}\rangle _{ABCD}= & {} \frac{1}{\sqrt{P_{2}^{(1)}}}(\alpha ^{2}|H\rangle _{A} |V\rangle _{C}|H\rangle _{B}|V\rangle _{D} +\beta ^{2}|V\rangle _{A}|H\rangle _{C}|V\rangle _{B}|H\rangle _{D})\nonumber \\&\otimes \gamma \delta (|l\rangle _{A}|l\rangle _{C}|r\rangle _{B}|r\rangle _{D} +|r\rangle _{A}|r\rangle _{C}|l\rangle _{B}|l\rangle _{D})\nonumber \\&\otimes \epsilon \varepsilon (|I\rangle _{A}|I\rangle _{C}|I\rangle _{B}|I\rangle _{D} +|E\rangle _{A}|E\rangle _{C}|E\rangle _{B}|E\rangle _{D}). \end{aligned}$$

Alice and Bob perform Hadamard operations on the three DOFs of photons C and D, respectively, and then the state $|\Psi _{2}\rangle _{ABCD}$ can be transformed into the state $|\Psi ^{\prime }_{2}\rangle _{ABCD}$. Here

15 $$\begin{aligned} |\Psi ^{\prime }_{2}\rangle _{ABCD}= & {} \frac{1}{\sqrt{2}}[(\alpha ^{(2)}|H\rangle _{A}|H\rangle _{B} +\beta ^{(2)}|V\rangle _{A}|V\rangle _{B}) |\phi ^{+}\rangle ^{P}_{CD}\nonumber \\&+(\alpha ^{(2)}|H\rangle _{A}|H\rangle _{B}-\beta ^{(2)}|V\rangle _{A}|V\rangle _{B}) |\psi ^{+}\rangle ^{P}_{CD}]\nonumber \\&\otimes \frac{1}{\sqrt{2}}(|\psi ^{+}\rangle ^{F}_{AB}|\phi ^{-} \rangle ^{F}_{CD}+|\psi ^{-}\rangle ^{F}_{AB}|\psi ^{-}\rangle ^{F}_{CD})\nonumber \\&\otimes \frac{1}{\sqrt{2}}(|\phi ^{+}\rangle ^{S}_{AB}|\phi ^{+} \rangle ^{S}_{CD}+|\phi ^{-}\rangle ^{S}_{AB}|\psi ^{+}\rangle ^{S}_{CD}).\nonumber \\ \end{aligned}$$

Here, $\alpha ^{(2)}=\frac{\alpha ^{2}}{\sqrt{|\alpha |^{4}+|\beta |^{4}}}$ and $\beta ^{(2)}=\frac{\beta ^{2}}{\sqrt{|\alpha |^{4}+|\beta |^{4}}}$. Then according to the measurement results in Table 2, we can obtain the state $|\psi _{2}^{(2)}\rangle _{AB}$ with or without single-photon operations, here

16 $$\begin{aligned} |\psi _{2}^{(2)}\rangle _{AB}= & {} (\alpha ^{(2)}|H\rangle _{A}|H\rangle _{B} +\beta ^{(2)}|V\rangle _{A}|V\rangle _{B}) \otimes |\psi ^{+}\rangle ^{F}_{AB}|\phi ^{+}\rangle ^{S}_{AB}. \end{aligned}$$

This is a partially hyperentangled Bell-type state with the longitudinal momentum DOFs in a maximally hyperentangled Bell state. For states $|\Psi _{3}\rangle _{ABCD}$ and $|\Psi _{4}\rangle _{ABCD}$, one can also obtain a partially hyperentangled Bell-type state with two DOFs in a maximally hyperentangled Bell state. The final state of photon pair AB can be denoted as

17 $$\begin{aligned} |\psi _{3}^{(2)}\rangle _{AB}= & {} |\phi ^{+}\rangle ^{P}_{AB}|\phi ^{+}\rangle ^{S}_{AB} \otimes (\gamma ^{(2)}|l\rangle _{A}|r\rangle _{B} +\delta ^{(2)}|r\rangle _{A}|l\rangle _{B}),\nonumber \\ |\psi _{4}^{(2)}\rangle _{AB}= & {} |\phi ^{+}\rangle ^{P}_{AB}|\psi ^{+}\rangle ^{F}_{AB} \otimes (\epsilon ^{(2)}|I\rangle _{A}|I\rangle _{B} +\varepsilon ^{(2)}|E\rangle _{A}|E\rangle _{B}). \end{aligned}$$

Here, $\gamma ^{(2)}=\frac{\gamma ^{2}}{\sqrt{|\gamma |^{4}+|\delta |^{4}}}$, $\delta ^{(2)}=\frac{\delta ^{2}}{\sqrt{|\gamma |^{4}+|\delta |^{4}}}$, $\epsilon ^{(2)}=\frac{\epsilon ^{2}}{\sqrt{|\epsilon |^{4}+|\varepsilon |^{4}}}$ and $\varepsilon ^{(2)}=\frac{\varepsilon ^{2}}{\sqrt{|\epsilon |^{4}+|\varepsilon |^{4}}}$. In this condition, another round of the hyper-ECP process is required.

In the third case, two of the parity check measurement results give odd-parity results. The four-photon system is projected into the state $|\Psi _{5}\rangle _{ABCD}$, $|\Psi _{6}\rangle _{ABCD}$ or $|\Psi _{7}\rangle _{ABCD}$. Then after the whole quantum circuit, Alice and Bob can get the two-photon system in the state $|\psi _{5}^{(2)}\rangle _{AB}$, $|\psi _{6}^{(2)}\rangle _{AB}$ or $|\psi _{7}^{(2)}\rangle _{AB}$ with or without single-photon operations. Here,

18 $$\begin{aligned} |\psi _{5}^{(2)}\rangle _{AB}= & {} (\alpha ^{(2)}|H\rangle _{A}|H\rangle _{B} +\beta ^{(2)}|V\rangle _{A}|V\rangle _{B}) \otimes (\gamma ^{(2)}|l\rangle _{A}|r\rangle _{B} +\delta ^{(2)}|r\rangle _{A}|l\rangle _{B}) \otimes |\phi ^{+}\rangle ^{S}_{AB},\nonumber \\ |\psi _{6}^{(2)}\rangle _{AB}= & {} (\alpha ^{(2)}|H\rangle _{A}|H\rangle _{B} +\beta ^{(2)}|V\rangle _{A}|V\rangle _{B}) \otimes (\epsilon ^{(2)}|I\rangle _{A}|I\rangle _{B} +\varepsilon ^{(2)}|E\rangle _{A}|E\rangle _{B}) |\psi ^{+}\rangle ^{F}_{AB},\nonumber \\ |\psi _{7}^{(2)}\rangle _{AB}= & {} (\gamma ^{(2)}|l\rangle _{A}|r\rangle _{B} +\delta ^{(2)}|r\rangle _{A}|l\rangle _{B}) \otimes (\epsilon ^{(2)}|I\rangle _{A}|I\rangle _{B} +\varepsilon ^{(2)}|E\rangle _{A}|E\rangle _{B}) \otimes |\phi ^{+}\rangle ^{P}_{AB}. \end{aligned}$$

The corresponding probabilities are shown in Table 1. For those partially hyperentangled states, another round of hyper-ECP is needed.

In the last case, all the three parity check measurements give odd-parity results. Then after the whole quantum circuit and conditional unitary operations on photon B, the two-photon system can be projected into the partially hyperentangled Bell-type state $|\psi _{8}^{(2)}\rangle _{AB}$ with the probability $P_{8}^{(1)}$. Here

19 $$\begin{aligned} |\psi _{8}^{(2)}\rangle _{AB}= & {} (\alpha ^{(2)}|H\rangle _{A}|H\rangle _{B} +\beta ^{(2)}|V\rangle _{A}|V\rangle _{B}) \otimes (\gamma ^{(2)}|l\rangle _{A}|r\rangle _{B} +\delta ^{(2)}|r\rangle _{A}|l\rangle _{B})\nonumber \\&\otimes (\epsilon ^{(2)}|I\rangle _{A}|I\rangle _{B} +\varepsilon ^{(2)}|E\rangle _{A}|E\rangle _{B}). \end{aligned}$$

This state can also be used in the next round to obtain the maximally hyperentangled Bell state.

For another identical four-photon system $\hbox {A}^{\prime }\hbox {B}^{\prime }\hbox {C}^{\prime }\hbox {D}^{\prime }$, the same operations are also performed on photon pairs $\hbox {A}^{\prime }\hbox {B}^{\prime }$ and $\hbox {C}^{\prime }\hbox {D}^{\prime }$ by Alice and Bob. Therefore, we can also obtain eight different collapsed states of photon pair $\hbox {A}^{\prime }\hbox {B}^{\prime }$, and the photon pair $\hbox {A}^{\prime }\hbox {B}^{\prime }$ in less-entangled state can be used as auxiliary photons in the next round.

*Improving the success probability by iteration* The success of the hyperconcentration schemes is based on the three parity checks. When three even-parity outcomes occur, the hyperconcentration schemes succeed with probability $P_{1}=P_{1}^{(1)}=8|\alpha \beta \gamma \delta \epsilon \varepsilon |^{2}$. Otherwise, these schemes fail. However, the other states can also be used to distill the maximally hyperentangled Bell state. In this subsection, we will use the auxiliary photon pair $\hbox {A}^{\prime }\hbox {B}^{\prime }$ to distill a maximally hyperentangled Bell state from the partially hyperentangled-type state obtained in the first round. That is, we will iterate the hyperentanglement concentration processes to improve the success probability, such method was first proposed in 2008^38^. The principle in the next round is similar to what is shown in Fig. 4.

For the second case, the partially hyperentangled Bell-type state is only less entangled in one DOF, while the other two DOFs are in the desired forms. Here, we only discuss the state $|\psi _{2}^{(2)}\rangle _{AB}$ in detail, while the other cases can be handled in the similar way. The state of the four-photon system ABA$^{\prime }$B$^{\prime }$ is $|\Psi _{2}^{1}\rangle _{ABA^{\prime }B^{\prime }}$, Here

20 $$\begin{aligned} |\Psi _{2}^{(2)}\rangle _{ABA^{\prime }B^{\prime }}= & {} |\psi _{2}^{(2)}\rangle _{AB}\otimes |\psi _{2}^{(2)}\rangle _{A^{\prime }B^{\prime }}\nonumber \\= & {} (\alpha ^{(2)}|H\rangle _{A}|H\rangle _{B}+\beta ^{(2)}|V\rangle _{A}|V\rangle _{B}) \otimes |\psi ^{+}\rangle ^{F}_{AB}|\phi ^{+}\rangle ^{S}_{AB}\nonumber \\&\otimes (\alpha ^{(2)}|H\rangle _{A^{\prime }}|H\rangle _{B^{\prime }} +\beta ^{(2)}|V\rangle _{A^{\prime }}|V\rangle _{B^{\prime }}) \otimes |\psi ^{+}\rangle ^{F}_{A^{\prime }B^{\prime }}|\phi ^{+}\rangle ^{S}_{A^{\prime }B^{\prime }}. \end{aligned}$$

In this case, we can just perform parity check for the less-entangled mode, hence Bob can let photons B and B$^{\prime }$ just pass through the P-QND. If the outcome of the P-QND is even, the two-photon system AB can be projected into the maximally hyperentangled Bell state $|\psi _{f}\rangle$, the success probability of this round is

21 $$\begin{aligned} P_{2}^{(2)}=P_{2}^{(1)}2|\alpha ^{(2)}\beta ^{(2)}|^{2} =P_{2}^{(1)}\frac{2|\alpha |^{4}|\beta |^{4}}{(|\alpha |^{4}+|\beta |^{4})^{2}}. \end{aligned}$$

For the partially hyperentangled Bell-type states $|\psi _{3}\rangle ^{1}_{AB}$ and $|\psi _{4}\rangle ^{1}_{AB}$, the success probabilities in the second round are $P_{3}^{(2)}$ and $P_{4}^{(2)}$, respectively. Here

22 $$\begin{aligned}&P_{3}^{(2)}=P_{3}^{(1)}2|\gamma ^{(2)}\delta ^{(2)}|^{2}=P_{3}^{(1)}\frac{2|\gamma |^{4}|\delta |^{4}}{(|\gamma |^{4}+|\delta |^{4})^{2}},\nonumber \\&P_{4}^{(2)}=P_{4}^{(1)}2|\epsilon ^{(2)}\varepsilon ^{(2)}|^{2}=P_{4}^{(1)}\frac{2|\epsilon |^{4}|\varepsilon |^{4}}{(|\epsilon |^{4}+|\varepsilon |^{4})^{2}}. \end{aligned}$$

Given the analysis of the second round for the second case, we can see that in the *m*th round, the probabilities of success (failure) of getting the desired state from the $(m-1)$th round are

23 $$\begin{aligned}&P^{(m)}_{2,s}=\frac{2|\alpha |^{2^{m}}|\beta |^{2^{m}}}{(|\alpha |^{2^{m}}+|\beta |^{2^{m}})^{2}},\;\; P^{(m)}_{2,f}=\frac{|\alpha |^{2^{m+1}}+|\beta |^{2^{m+1}}}{(|\alpha |^{2^{m}}+|\beta |^{2^{m}})^{2}},\nonumber \\&P^{(m)}_{3,s}=\frac{2|\gamma |^{2^{m}}|\delta |^{2^{m}}}{(|\gamma |^{2^{m}}+|\delta |^{2^{m}})^{2}},\;\; P^{(m)}_{3,f}=\frac{|\gamma |^{2^{m+1}}+|\delta |^{2^{m+1}}}{(|\gamma |^{2^{m}}+|\delta |^{2^{m}})^{2}},\nonumber \\&P^{(m)}_{4,s}=\frac{2|\epsilon |^{2^{m}}|\varepsilon |^{2^{m}}}{(|\epsilon |^{2^{m}}+|\varepsilon |^{2^{m}})^{2}},\;\; P^{(m)}_{4,f}=\frac{|\epsilon |^{2^{m+1}}+|\varepsilon |^{2^{m+1}}}{(|\epsilon |^{2^{m}}+|\varepsilon |^{2^{m}})^{2}}.\nonumber \\ \end{aligned}$$

Here $P^{(m)}_{i,s}$ and $P^{(m)}_{i,f}$ (i = 2,3,4) denote the success and failure probabilities for obtaining the maximally hyperentangled state in the *m*th round, respectively. Then we can compute the success probability $P_{i}$ (i = 2,3,4) after *n* $(n>2)$ rounds for the second case,

24 $$\begin{aligned} P_{i}= & {} P_{i}^{(1)}(P_{i,s}^{(2)}+P_{i,f}^{(2)}P_{i,s}^{(3)} +\cdots +P_{i,f}^{(2)}P_{i,f}^{(3)}\cdots P_{i,f}^{(n-1)}P_{i,s}^{(n)}). \end{aligned}$$

For the third case, the partially hyperentangled Bell-type state is less entangled in two DOFs, while the third DOF is in maximally entangled state. Thus, in the second round, we will pay attention to two QNDs. Here, we will discuss the state $|\psi _{5}^{(2)}\rangle _{AB}$ in detail, the other cases can be handled in the similar way. If the photons B and B$^{\prime }$ are in even-parities in polarization and the first longitudinal momentum DOF, we can obtain the maximally hyperentangled Bell state $|\psi _{f}\rangle$ with the probability

25 $$\begin{aligned} P_{5}^{(2)}= & {} P_{5}^{(1)}P_{5ee}^{(2)}=P_{5}^{(1)}4|\alpha ^{(2)}\beta ^{(2)}\gamma ^{(2)}\delta ^{(2)}|^{2} =P_{5}^{(1)}P_{2,s}^{(2)}P_{3,s}^{(2)}. \end{aligned}$$

For the state $|\psi _{5}^{(m)}\rangle _{AB}$ which corresponds to two odd-parity results in the $(m-1)$th round, the probabilities of the four parity check results are

26 $$\begin{aligned}&P_{5ee}^{(m)}=P_{2,s}^{(m)}P_{3,s}^{(m)},\;\;\;\; P_{5eo}^{(m)}=P_{2,s}^{(m)}P_{3,f}^{(m)},\;\;\;\; P_{5oe}^{(m)}=P_{2,f}^{(m)}P_{3,s}^{(m)},\;\;\;\; P_{5oo}^{(m)}=P_{2,f}^{(m)}P_{3,f}^{(m)}. \end{aligned}$$

The subscripts “ee”, “eo”, “oe” and “oo” indicate the parity check results for two QNDs, with “e” being even and “o” being odd. In this case, the success probability of the *m*th $(m>2)$ round is

27 $$\begin{aligned} P_{5}^{(m)}= & {} P_{5}^{(1)}(P_{2,s}^{(2)}+P_{2,f}^{(2)}P_{2,s}^{(3)}+\cdots +P_{2,f}^{(2)}P_{2,f}^{(3)}\cdots P_{2,f}^{(m-2)}P_{2,s}^{(m-1)})P_{3,f}^{(2)}P_{3,f}^{(3)}\cdots P_{3,f}^{(m-1)}P_{3,s}^{(m)}\nonumber \\&+P_{5}^{(1)}(P_{3,s}^{(2)}+P_{3,f}^{(2)}P_{3,s}^{(3)}+\cdots +P_{3,f}^{(2)}P_{3,f}^{(3)}\cdots P_{3,f}^{(m-2)}P_{3,s}^{(m-1)})P_{2,f}^{(2)}P_{2,f}^{(3)}\cdots P_{2,f}^{(m-1)}P_{2,s}^{(m)}\nonumber \\&+P_{5}^{(1)}P_{2,f}^{(2)}P_{3,f}^{(2)}P_{2,f}^{(3)}P_{3,f}^{(3)}\cdots P_{2,f}^{(m-1)}P_{3,f}^{(m-1)}P_{2,s}^{(m)}P_{3,s}^{(m)}. \end{aligned}$$

We can easily achieve the total success probability after *n* $(n>2)$ rounds

28 $$\begin{aligned} P_{5}=\sum _{m=2}^{n}P_{5}^{(m)}. \end{aligned}$$

For the partially hyperentangled Bell-type states $|\psi _{6}^{(2)}\rangle _{AB}$ and $|\psi _{7}^{(2)}\rangle _{AB}$, the corresponding success probabilities are

29 $$\begin{aligned} P_{6}^{(2)}= & {} P_{6}^{(1)}P_{6ee}^{(2)}=P_{6}^{(1)}4|\alpha ^{(2)}\beta ^{(2)}\epsilon ^{(2)}\varepsilon ^{(2)}|^{2} =P_{6}^{(1)}P_{2,s}^{(2)}P_{4,s}^{(2)},\nonumber \\ P_{7}^{(2)}= & {} P_{7}^{(1)}P_{7ee}^{(2)}=P_{7}^{(1)}4|\gamma ^{(2)}\delta ^{(2)}\epsilon ^{(2)}\varepsilon ^{(2)}|^{2} =P_{7}^{(1)}P_{3,s}^{(2)}P_{4,s}^{(2)}.\nonumber \\ \end{aligned}$$

For the partially hyperentangled Bell-type states $|\psi _{6}^{(m)}\rangle _{AB}$ and $|\psi _{7}^{(m)}\rangle _{AB}$, the probabilities of the four parity check results are

30 $$\begin{aligned}&P_{6ee}^{(k)}=P_{2,s}^{(m)}P_{4,s}^{(m)},\;\;\;\; P_{6eo}^{(k)}=P_{2,s}^{(m)}P_{4,f}^{(m)},\;\;\;\; P_{6oe}^{(k)}=P_{2,f}^{(m)}P_{4,s}^{(m)},\;\;\;\; P_{6oo}^{(k)}=P_{2,f}^{(m)}P_{4,f}^{(m)},\nonumber \\&P_{7ee}^{(k)}=P_{3,s}^{(m)}P_{4,s}^{(m)},\;\;\;\; P_{7eo}^{(k)}=P_{3,s}^{(m)}P_{4,f}^{(m)},\;\;\;\; P_{7oe}^{(k)}=P_{3,f}^{(m)}P_{4,s}^{(m)},\;\;\;\; P_{7oo}^{(k)}=P_{3,f}^{(m)}P_{4,f}^{(m)}. \end{aligned}$$

The corresponding success probabilities of the *m*th $(m>2)$ round are

31 $$\begin{aligned} P_{6}^{(m)}= & {} P_{6}^{(1)}(P_{2,s}^{(2)}+P_{2,f}^{(2)}P_{2,s}^{(3)}+\cdots +P_{2,f}^{(2)}P_{2,f}^{(3)}\cdots P_{2,f}^{(m-2)}P_{2,s}^{(m-1)})P_{4,f}^{(2)}P_{4,f}^{(3)}\cdots P_{4,f}^{(m-1)}P_{4,s}^{(m)}\nonumber \\&+P_{6}^{(1)}(P_{4,s}^{(2)}+P_{4,f}^{(2)}P_{4,s}^{(3)}+\cdots +P_{4,f}^{(2)}P_{4,f}^{(3)}\cdots P_{4,f}^{(m-2)}P_{4,s}^{(m-1)})P_{2,f}^{(2)}P_{2,f}^{(3)}\cdots P_{2,f}^{(m-1)}P_{2,s}^{(m)}\nonumber \\&+P_{6}^{(1)}P_{2,f}^{(2)}P_{4,f}^{(2)}P_{2,f}^{(3)}P_{4,f}^{(3)}\cdots P_{2,f}^{(m-1)}P_{4,f}^{(m-1)}P_{2,s}^{(m)}P_{4,s}^{(m)},\nonumber \\ P_{7}^{(m)}= & {} P_{7}^{(1)}(P_{3,s}^{(2)}+P_{3,f}^{(2)}P_{3,s}^{(3)}+\cdots +P_{3,f}^{(2)}P_{3,f}^{(3)}\cdots P_{3,f}^{(m-2)}P_{3,s}^{(m-1)})P_{4,f}^{(2)}P_{4,f}^{(3)}\cdots P_{4,f}^{(m-1)}P_{4,s}^{(m)}\nonumber \\&+P_{7}^{(1)}(P_{4,s}^{(2)}+P_{4,f}^{(2)}P_{34,s}^{(3)}+\cdots +P_{34,f}^{(2)}P_{4,f}^{(3)}\cdots P_{4,f}^{(m-2)}P_{4,s}^{(m-1)})P_{3,f}^{(2)}P_{3,f}^{(3)}\cdots P_{3,f}^{(m-1)}P_{3,s}^{(m)}\nonumber \\&+P_{7}^{(1)}P_{3,f}^{(2)}P_{4,f}^{(2)}P_{3,f}^{(3)}P_{4,f}^{(3)}\cdots P_{3,f}^{(m-1)}P_{4,f}^{(m-1)}P_{3,s}^{(m)}P_{4,s}^{(m)}. \end{aligned}$$

By iterating the hyperconcentration process *n* $(n>2)$times, the total success probability for each condition is

32 $$\begin{aligned} P_{6}=\sum _{m=2}^{n}P_{6}^{(m)},\;\;\;\;P_{7}=\sum _{m=2}^{n}P_{7}^{(m)}. \end{aligned}$$

For the last case, the principle of this step is the same as the first round, except that the photon pair CD is replaced by $\hbox {A}^{\prime }\hbox {B}^{\prime }$. Thus, using the whole quantum circuit shown in Fig. 4, we can achieve the maximally hyperentangled Bell state $|\psi _{f}\rangle$ with the probability

33 $$\begin{aligned} P_{8}^{(2)}= & {} P_{8}^{(1)}8|\alpha ^{(2)}\beta ^{(2)}\gamma ^{(2)} \delta ^{(2)}\epsilon ^{(2)}\varepsilon ^{(2)}|^{2}=P_{8}^{(1)}P_{2,s}^{(2)}P_{3,s}^{(2)}P_{4,s}^{(2)}. \end{aligned}$$

The success probability of the 3th round is

34 $$\begin{aligned} P_{8}^{(3)}= & {} P_{8}^{(1)}(P_{3,s}^{(2)}P_{4,s}^{(2)}P_{2,f}^{(2)}P_{2,s}^{(3)}+P_{2,s}^{(2)}P_{4,s}^{(2)}P_{3,f}^{(2)}P_{3,s}^{(3)} +P_{2,s}^{(2)}P_{3,s}^{(2)}P_{4,f}^{(2)}P_{4,s}^{(3)}\nonumber \\&+P_{2,f}^{(2)}P_{3,f}^{(2)}P_{4,s}^{(2)}P_{2,s}^{(3)}P_{3,s}^{(3)} +P_{2,f}^{(2)}P_{4,f}^{(2)}P_{3,s}^{(2)}P_{2,s}^{(3)}P_{4,s}^{(3)}+P_{3,f}^{(2)}P_{4,f}^{(2)}P_{2,s}^{(2)}P_{3,s}^{(3)}P_{4,s}^{(3)}\nonumber \\&+P_{2,f}^{(2)}P_{3,f}^{(2)}P_{4,f}^{(2)}P_{2,s}^{(3)}P_{3,s}^{(3)}P_{4,s}^{(3)}). \end{aligned}$$

Then we can obtain the success probability of the *m*th $(m>3)$ round for the last case

35 $$\begin{aligned} P_{8}^{(m)}= & {} P_{8}^{(1)}\sum _{a,b,c}[(P_{a,s}^{(2)}P_{b,s}^{(2)}+P_{a,f}^{(2)}P_{b,f}^{(2)}P_{a,s}^{(3)}P_{b,s}^{(3)}\nonumber \\&+\cdots +P_{a,f}^{(2)}P_{b,f}^{(2)}\cdots P_{a,f}^{(m-2)}P_{b,f}^{(m-2)}P_{a,s}^{(m-1)}P_{b,s}^{(m-1)}) P_{c,f}^{(2)}\cdots P_{c,f}^{(m-1)}P_{c,s}^{(m)}\nonumber \\&+(P_{c,s}^{(2)}+P_{c,f}^{(2)}P_{c,s}^{(3)}+\cdots +P_{c,f}^{(2)}P_{c,f}^{(3)}\cdots P_{c,f}^{(m-2)}P_{c,s}^{(m-1)})P_{a,f}^{(2)}P_{b,f}^{(2)}\cdots P_{a,f}^{(m-1)}P_{b,f}^{(m-1)}P_{a,s}^{(m)}P_{b,s}^{(m)}]\nonumber \\&+P_{8}^{(1)}\sum _{i,j,k}\left[ \sum _{l=2}^{m-2}(P_{i,s}^{(2)}+P_{i,f}^{(2)}P_{i,s}^{(3)}\right. \nonumber \\&\left. +\cdots +P_{i,f}^{(2)}P_{i,f}^{(3)}\cdots P_{i,f}^{(l-1)}P_{i,s}^{(l)})P_{j,f}^{(2)}\cdots P_{j,f}^{(l)}P_{j,s}^{(l+1)}\right] P_{k,f}^{(2)}\cdots P_{k,f}^{(m-1)}P_{k,s}^{(m)}\nonumber \\&+P_{8}^{(1)}P_{2,f}^{(2)}P_{3,f}^{(2)}P_{4,f}^{(2)}\cdots P_{2,f}^{(m-1)}P_{3,f}^{(m-1)}P_{4,f}^{(m-1)}P_{2,s}^{(m)}P_{3,s}^{(m)}P_{4,s}^{(m)}. \end{aligned}$$

Here, $a\ne b\ne c\in \{2, 3, 4\}$ and satisfy the limitation $a<b$, while $i\ne j\ne k\in \{2, 3, 4\}$.

The total success probability for the last case is

36 $$\begin{aligned} P_{8}=\sum _{m=2}^{n}P_{8}^{(m)}. \end{aligned}$$

Through *n* rounds of our hyper-ECP, the success probability can be improved as

37 $$\begin{aligned} P=P_{1}+P_{2}+P_{3}+P_{4}+P_{5}+P_{6}+P_{7}+P_{8}. \end{aligned}$$

**Figure 5:**
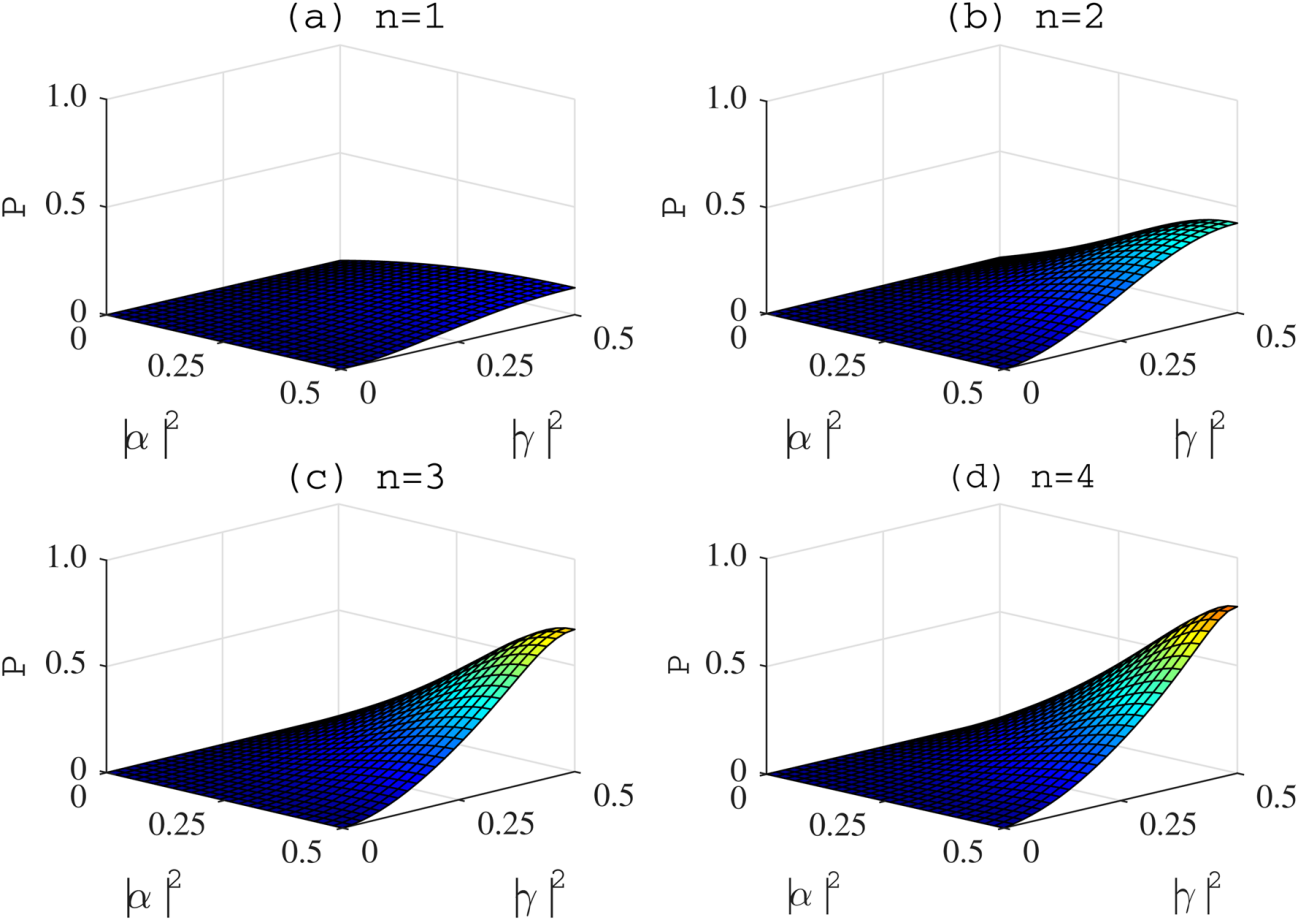
The success probability P of our hyper-ECP for two-photon six-qubit systems in an arbitrary partially hyperentangled Bell state. The parameters of the arbitrary partially hyperentangled Bell states are chosen as $|\gamma |=|\epsilon |$. $n=i$ ($i=1, 2, 3, 4$) is the iteration number. Different figures correspond to the schemes with different number of iterations.

The relation between the total success probability, the initial parameter and the iteration number is shown in Fig. 5. The four plots show the success probabilities for $n = 1,2,3,4$ iterations. The parameters of the arbitrary partially hyperentangled Bell states are chosen as $|\gamma |=|\epsilon |$. As shown in Fig. 5, we can find that, the total success probability is correlated to the six parameters of the partially hyperentangled Bell state, and with the iteration of our hyper-ECP process, the success probability *P* will be greatly improved. The maximum of the success probability can reach $95.4\%$ after $n=6$ iterations, and it can be improved to nearly $100\%$ by iteration.

## Discussion

We have proposed an efficient hyper-ECP for partially hyperentangled Bell states in the polarization and double longitudinal momentum DOFs resorting to the cross-Kerr nonlinearity. We focus on the case that the parameters of the partially hyperentangled Bell state are unknown to the two legitimate users. In our hyper-ECP, two identical partially hyperentangled pairs are used to distill maximally hyperentangled Bell state. Hyperconcentration is realized with the three parity checks, where one for the polarization state and the others two are for double longitudinal momentum mode. Both the users are required to perform some quantum operations on their photons. After the first hyperconcentration round, the protocol has a certain success probability $P_{1}$, and the success probability can be greatly improved by implementing more rounds of hyperconcentration.

In our hyper-ECP, the cross-Kerr nonlinearity is developed to structure the QNDs to check the parity of the two-photon in three DOFs. Thus, it will affect the practical efficiency of our hyper-ECP. Although a lot of works have been reported on cross-Kerr nonlinearity^58,59^, we should acknowledge that the cross-Kerr effect in the single-photon regime is a big challenge with current technology^60–62^. However, recent researches show that the use of cross-Kerr effect is promising in the not so distant future^63–72^. For example, in 2011, He *et al.*^63^ researched the interaction between continuous-mode coherent states and single photons, and they indicated that the effects influence the cross-phase modulation process and let the treatment of single-photon-coherent-state interactions more practical. In the same year, Feizpour *et al.*^64^ showed that a cross-Kerr phase shift is capable of being amplified to an observable value by using weak-value amplification. In 2016, Beck *et al.*^70^ achieved a large conditional cross-phase shift of $\pi /6$ between a retrieved signal and control photons. In the same year, Tiarks *et al.*^71^ demonstrated the generation of a $\pi$ phase shift with a single-photon pluse. In 2019, Sinclair *et al.*^72^ reported strong cross-phase modulation at low light levels generated by a resonant cross-Kerr nonlinearity based on Rydberg interactions and electromagnetically induced transparency. The nonlinear phase was measured as large as 8 mrad per nanowatt of signal power, corresponding to a $\chi ^{(3)}$ of $10^{-8} m^{2}/V^{2}$. On the other hand, in our scheme, a small phase shift, as long as it can be discriminated from zero, is required. All those researches indicate that our scheme is possible to be realized with the current experimental techniques. Moreover, other kinds of nonlinear interaction can also provide practicable ways to realize the needed parity check.

The hyperconcentration of the unknown partially hyperentangled Bell states in our scheme can also be achieved using linear optics. However, the restriction on hyperconcentration schemes based on linear optics is that they can not iterate and the success probabilitis are lower than those with nonlinear optics. Certainly, we can first perform parameter estimation and then use the hyperconcentration schemes for states with known parameters to get higher success probability. But in fact, the estimation of parameters consumes extra quantum resources. In our scheme, we can extract all the useful entanglement source, and the success probability can reach the theory limit with the help of iteration. Although the success probability of our scheme can be greatly increased, this scheme can only distill the maximal hyperentangled Bell states from less entangled pure states. For other kinds of decoherence, further research is required.

In summary, we have proposed an efficient hyper-ECP for partially hyperentangled states that are entangled in polarization and double longitudinal momentum DOFs. In our hyper-ECP, the partially hyperentangled states have six arbitrary parameters, which are unknown to the two legitimate users, and the QNDs resorting to the cross-Kerr nonlinearity are used to accomplish our scheme. The unsuccessful instances in each round can be reconcentrated to achieve higher success probability. Since hyperentanglement has many potential applications, our hyper-ECP may be useful in long-distance quantum communication in the future.

## Methods

### Cross-Kerr nonlinearity

The Hamiltonian of a cross-Kerr nonlinearity medium is^58,73^ $H=\hbar \chi a^{\dag }_{s}a_{s}a^{\dag }_{p}a_{p}$. $a^{\dag }_{s}$ ($a^{\dag }_{p}$) and $a_{s}$ ($a_{p}$) are the creation and the annihilation operators of the signal (probe) pulse beam, respectively. $\hbar \chi$ is the coupling strength of the nonlinearity, which depends on the nonlinear material. If the probe beam is in the coherent state $|\alpha \rangle$, for a single photon $|\varphi \rangle _{s}=c_{0}|0\rangle _{s}+c_{1}|1\rangle _{s}$, the effect of the cross-Kerr nonlinearity on the whole system can be denoted as

38 $$\begin{aligned} U|\varphi \rangle _{s}|\alpha \rangle _{p}= & {} e^{iHt/\hbar }(c_{0}|0\rangle _{s} +c_{1}|1\rangle _{s})|\alpha \rangle _{p}\nonumber \\= & {} c_{0}|0\rangle _{s}|\alpha \rangle _{p}+c_{1}|1\rangle _{s}|\alpha e^{i\theta } \rangle _{p}, \end{aligned}$$

where $|0\rangle _{s}$ and $|1\rangle _{s}$ are the Fock states for the signal pulse, and *t* is the interaction time. The phase shift $\theta =\chi t$ is proportional to the number of photons with the single-photon state being unaffected.
